# Speaking Out on Sexualized Violence Through Artistic Storytelling in Post-conflict Northern Ireland

**DOI:** 10.1177/10778012231179222

**Published:** 2023-06-06

**Authors:** Anna-Lea Van Ooijen, Tine Davids, Lisa Fitzpatrick

**Affiliations:** 1 Hogeschool Utrecht /University Medical Hospital, Utrecht, The Netherlands; 2 6029 Radboud University, Nijmegen, The Netherlands; 3 Ulster university, Derry-Londonderry, Northern Ireland

**Keywords:** sexualized violence, artistic storytelling, testimonial theater, Northern Ireland

## Abstract

Directly related to The Troubles, sexualized violence has been largely ignored, yet continues to affect significant numbers of individuals in Northern Ireland today. This article analyzes various women's stories concerning sexualized violence shared in testimonial theater projects in Northern Ireland. We argue that (a) artistic storytelling about sexualized violence in theater projects can be a tool to release individuals and the collective from the (normalized) silence surrounding these violent acts and (b) it is an epistemological transformative method of inquiry to address these acts with the aim of eliminating them.

## Introduction

With the global upsurge of the #MeToo movement, and increasing numbers of research reports charting the ubiquity of sexual harassment in public spaces (Anyadike-Danes et al., 2022), sexual violence has become a recognized societal problem and has signaled a call for change. And yet, many individuals of all genders find themselves unable to tell their stories, feeling stigmatized, ashamed, misunderstood, and ignored against an existing culture of impunity and silence. It is then crucial to understand how speaking about sexual violence can work and spur meaningful societal change.

Several studies point to the importance of storytelling in addressing sexual violence. The majority of feminist scholars, however, have focused on traditional and conventional forms of storytelling and what these can do to address the violence (e.g., Gay, 2019; Kelly, 2013; Serisier, 2020; Wieskamp, 2018): examples include rape memoirs and rape recovery memoirs (e.g., Angelou, 2010; Bowdler, 2020; Brison, 2002; Kalven, 1999; Sebold, 2019). Therefore, this article will explore artistic storytelling in testimonial theater productions as an alternative and creative way of storytelling about sexual violence to address the violence with the aim of eliminating it. With testimonial theater, we refer to the act of testifying on stage about a traumatic event (Waterson, 2010).

This article draws on the experiences of women participants of theater projects in post-conflict Northern Ireland with sexual abuse. In Northern Ireland, sexual violence is a pressing social issue (Green, 2019). Some women spoken to for this study even suggest that likely every second Northern Irish girl experiences sexual violence (Anyadike-Danes et al., 2022). The pervasiveness of sexual violence in Northern Ireland needs to be understood against the backdrop of the Northern Irish conflict, widely known as The Troubles, which was a 30-year sectarian conflict (1969 to 1998) between Protestant Loyalist (Unionist) and Catholic Republican (Nationalist) communities (Cairns and Darby, 1998). Northern Irish society was profoundly “patriarchal and embedded within conservative discourses” (Cockburn, 1998, p. 58). The conflict is therefore commonly referred to as an “armed patriarchy” (The Guardian, 2021, sec. 14). This meant that Northern Ireland was a disputed region, fought over by armed men: agents of the state, as well as agents of paramilitary groups. All of these actors: the state, its forces, and the paramilitaries were overwhelmingly masculine and were acting to preserve or institute versions of the patriarchal state.

Although the conflict has ended, the sectarian divide remains (Nolan, 2014), resulting in occasional riots, and reemerging strongly as intercommunity tension following the Brexit vote and ongoing disruption. Northern Ireland is argued to be in a state of imperfect peace (Napier et al., 2017; Neumann, 2002), referring to peace as not absolute or perpetual, but a process along an unfinished road (Munoz, 2006). It is against the background of the conflict and the continuing struggle for peace that men's dominance and power underlie the violent reality of women's daily lives in Northern Ireland (Gilmartin, 2018). So far, Northern Ireland has no integrated strategy to tackle sexual violence, and perpetrators of the violence are rarely convicted. As a result, as indicated by O’Rourke and Swaine (2017), violence against women remains underdocumented and underreported, and is a story about Northern Ireland “yet to be told.”

The focus in this article on women's testimonies of violence is based on the performances studied. We emphasize that people of all gender fall victim to sexual violent acts. We note that not only women suffer under patriarchy, men and all other genders suffer too, albeit in different ways (see for instance Zarkov, 2001). The findings presented in this study are based on data gathered by the first author in Derry-Londonderry Northern Ireland during a three-month fieldwork period in early 2020, which focused on conflict narratives shared through testimonial theater, and highlight this form of storytelling as a navigational tool to write peace into the future. In total, 18 respondents participated in this study, with whom interviews were conducted and different participatory methods (e.g., participant observation and mapping exercises) were applied.

The study involved 12 participants of Theatre of Witness productions and a Theatre and Peacebuilding Academy production, both facilitated by the Derry Playhouse Theatre (these projects will be discussed later in the article). Moreover, interviews were held with artistic directors and facilitators of the different theater projects, and researchers from Ulster University in Derry-Londonderry were contacted to discuss the topic of the study. The researcher also participated in various community workshops about the theater projects organized by the Derry Playhouse.

For this article, follow-up interviews were conducted in late 2020 and early 2021 with five women participants of the theater projects to reflect solely on their experiences with sexual violence and reflect on testimonial theater as a means to speak up about the violence. Additionally, we interviewed the artistic director of Kabosh Theatre Company in Belfast and were present during a roundtable discussion of one of their recent productions that addresses sexual violence in times of conflict (more information about this theater company and the production mentioned will follow). We want to emphasize that all respondents gracefully shared their stories with us and gave consent for their stories to be shared for academic purposes.

The findings presented in this article are thus based upon empirical engagement and literature review, as we analyze the testimonies of sexual violence from the women participants of the theater projects and examine their experiences of partaking in those projects. The article is organized as follows. It first provides an overview of the theater projects and the women whose stories we outline. After that, we conceptualize the term *sexualized violence*, which we will use throughout the text, replacing the term sexual violence. And by outlining the stories of the women participants in the theater projects, we illustrate the violence as sexualized and interpret it against the patriarchal and conflictual background. With this in mind, we turn to the three main findings presented in this study. The first finding considers artistic storytelling in the theater as transformative on an individual level. The second finding examines artistic storytelling in the theater as transformative on an interpersonal and wider societal level. For these findings, we consider the concept of *safe space* and the *personal truth* of the shared narratives as key to individual and social transformation. The last finding discusses artistic storytelling in the theater as a method of research and a social intervention. We then identify the limits and risks of artistic storytelling in the theater regarding sexual violence. At last, a critical analysis is provided about what artistic storytelling on sexual violence in the theater can do to speak out about and against the violence.

## The Theater Projects and the Research Participants

We discuss three theater projects in this article: the Theatre of Witness, the Theatre and Peacebuilding Academy, and the work of Kabosh Theatre Company. The Theatre of Witness is a model of performance aimed at social transformation. It was founded by a dancer and therapist, Teya Sepinuck, and is made up of a series of preshow workshops that entailed collective storytelling, writing scripts, rehearsals, performances, after-show discussions, and workshops in various communities in Northern Ireland. The Theatre of Witness productions worked with first-person narratives about life during the conflict and its aftermath, creating performances where participants speak of their own experiences before a live audience (Weiglhofer, 2014). As Sepinuck describes it,

The purpose of this form of theater is to give voice to those who have been marginalized, forgotten, or are invisible in the larger society, and to invite audiences to bear witness to issues of suffering, redemption, and social justice (Sepinuck, 2013, p. 14).

Minimalist theatrical aesthetics were used: filmed material is projected onto the back of the playing area, simple lighting and some background music are used, and very few props. In addition to the productions, community workshops took place where “two or three performers met with youth and (other) community groups to engage in discussion, sometimes after excerpts of the performance were shown on film” (Weiglhofer, 2014, p. 17).

Various Theatre of Witness productions were facilitated by the Derry Playhouse in Derry-Londonderry between 2009 and 2018. These productions were funded by the Special European Union Programmes Body (SEUPB), which funds a wide range of projects for peacebuilding. Through the different testimonial performances seeds for change were sown, as Grant explains: “the Theatre of Witness […] seeks to eschew labels such as victim, survivor and perpetrator, and […] allow[s] sometimes contradictory and unreconciled accounts of the region's recent history to sit together side by side, shoulder to shoulder” (2016, p. 2). We focus on the show We Carried Your Secrets in 2009, which explored transgenerational trauma, and I Once Knew a Girl in 2010, which is an all-women production.

Like the Theatre of Witness, the later Theatre and Peacebuilding Academy was facilitated by the Derry Playhouse and funded by the SEUPB. It also used first-person staged narratives acted out by the people themselves live on stage. Our focus is on the show First Response in 2020, which explored the experiences of members of the emergency services during the conflict. Another company whose projects work with the legacies of the Northern Irish conflict through storytelling in the theater is Kabosh Theatre Company. Kabosh uses professional actors to act out authored plays that draw upon real-person stories on live theatrical stages. This work is closer to a professional theater aesthetic than the testimonial form of the other projects. One of their recent productions The Shedding of Skin premiering in 2021 draws upon stories from the Ancient Greek tragedy, to World War II, to more recent reports of abusive atrocities in the Balkans and other parts of the world. We see many of the experiences and stories of the Northern Irish women reflected in this production.

This article centers around the stories of five women from different generations who participated in a Theatre of Witness or Theatre and Peacebuilding Academy project. Although there is a risk of overexposing and exploiting, and/or thriving on the misery of others for academic purposes, all participants bravely shared their stories with us and gave consent for their stories to be integrated into this article. They emphasized the need to speak openly about sexualized violence to break the silence, despite their hesitation about the shame attached to victims of such violence and fear of exposure. Despite the risks involved, some participants explicitly wanted their stories to be told to raise awareness about the issue and find effective solutions. Due to the sensitivity of the topic, all names of the participants are anonymized, except for Anne, as per their wishes.

Three women took part in Theatre of Witness productions. Mary (daughter of a policeman) was a part of the production We Carried Your Secrets in 2009, Anne (a former Irish Republican Army [IRA] combatant) and Karen (a woman from a family displaced by the conflict) took part in the production I Once Knew a Girl in 2010. Anne and Karen have adult experience of the conflict, while Mary's experience was as a child. The other two women, Aileen and Jane, were participants in the Theatre and Peacebuilding Academy production First Response production in 2020. These women are in their early adulthood and part of the post-conflict generation. Except for the Kabosh Theatre production, the different Theatre of Witness and Theatre and Peacebuilding Academy productions and community workshops did not center around the topic of sexualized violence, yet stories of abuse came to the fore as the women spoke about their lives during the conflict and its aftermath. They raised issues of sexual violence as pressing and urgent.

## Conceptualizing Sexualized Violence

This article draws on the notion of sexual violence or sexual abuse, which we use interchangeably. Sexual abuse pertains to the pervasive social problem that concerns coerced forms of sexual behaviors, often with a profound impact on the physical and mental health of survivors of all genders. Sexual violence, as defined by the Krug et al. (2002) quoting the WHO definition of the violence:

Any sexual act, attempt to obtain a sexual act, unwanted sexual comments or advances, or acts to traffic, or otherwise directed, against a person's sexuality using coercion, by any person regardless of their relationship to the victim, in any setting, including but not limited to home and work (p.149).

As often stated by researchers, sexual violence is gender-based and rooted in asymmetrical power relations that associate masculinity with power, control, and dominance, and femininity with weakness, passivity, and submissiveness. As explained earlier, in Northern Ireland gendered relations are profoundly patriarchal (Cockburn, 1998). Such patriarchal discourses produce and normalize acceptable codes on men's and women's behavior, rendering femininity and masculinity as dichotomous, justifying and reinforcing a heteronormative ideology. Moreover, sexual violence against all genders is known to be a weapon of war and conflict in constructing ethnic/national/religious and other boundaries between groups via the consolidation of heteronormativity and hegemonic masculinity (see, among others, for instance: Zarkov, 2001). In outlining the stories of the Northern Irish participants of the theater projects, this article particularly focuses on women's experiences of sexual violence against the persistent heteronormative gender discourses.

It needs to be mentioned that, while in our description and analysis, the terminology of men and women may come to the fore, this is not meant to underscore a binary vision on gender. Instead, we recognize that sexualized violence against all genders requires much attention and research. Yet, for the purpose of this article, we limit our scope, based on our research material, only to women's experiences.

Although sexual violence is the generic term used in academic studies, we are inspired by Kloß's (2016) work, which refers to sexual(ized) violence to “emphasize that the core of this behavior lies not in a sexual attraction but modes of reinforcing (patriarchal) power” (Kloß, 2016, p. 400). It defines the violence as a social mechanism of control that reasserts and reaffirms masculine power, whether in times of conflict or of imperfect peace. This is also pointed out by Hume (2009), who considers sexual abuse as normalized and rationalized under “accepted codes” of masculine behavior and discourses on masculinity (p. 4). We employ Kloß's (2016) term “sexualized violence” (erasing the brackets) and understand the violence as an almost ordinary aspect of social life that feeds into a culture of impunity and silence, that is, fueled by discourses that articulate fear, shame, and stigma in light of uneven gendered power relations.

Sexualized violence during armed conflict, but also during its aftermath, builds on and exaggerates ordinary and everyday discourses of gendered power relations. In particular, in a conflict-ridden or segregated society talking about sexualized violence tends to incur shame and stigma and can be hazardous because the speaker may be accused of crossing group boundaries, and conspiring with the enemy. Inspired by Hume (2009), we see the testimonies of violence as everyday epistemologies of sexualized violence, reframing sexual violence as something that is situated in immediate and familiar places, something that people of all genders may be confronted with in a conflict-torn society. Next, we illustrate how the normalization of everyday sexual abuse gets articulated in the language of The Troubles and its aftermath, and vice versa sexual abusers feed on the already existing normalization of sexualized violence.

## Sexualized Violence in the Personal Testimonies of the Northern Irish Women

The theme of masculinity and war can be found in the personal testimonies of sexualized violence of the women participants of the various theater projects. To make this intelligible we outline and analyze the stories of Karen and Anne, who were participants of the Theatre of Witness production called I Once Knew a Girl, staged in 2010. Karen was born in a displaced republican family. Her family home functioned as a safe house for the IRA; there was no privacy. Karen first experienced sexual abuse at the age of six by her cousin. He had told her to come with him to their grandparents’ house, however, no one was home. She had to play a game with him, and it was then that he abused her. He told her to keep quiet. In the years that followed Karen was abused by different men. She was about nine years old when the babysitter from church abused her. At the age of 10 or 11, she was abused by her uncle, who had come live with the family during the conflict. During the theater production, she explains that one time while she was making breakfast her uncle, who lay smoking and drinking on the couch of their home, whispered in her ear, “if you let me touch you, I give you money.” But, according to Karen, no one ever saw anything untoward happen. Once married, Karen endured a seven-year-long violent relationship with her husband. This relationship isolated her, resulting in severe mental health problems, such as loss of self-esteem, depression, and suicidal thoughts. She had tried counseling, but could not speak.

Anne, who partook in the Theatre of Witness production I Once Knew a Girl in 2010, shares a similar experience with Karen. Although most women were confined to the private spheres of the household (Green, 2019), Anne joined the IRA^1^ and worked as a quartermaster. Anne was therefore a part of a minority of women who became actively involved in the political realm (McDowell, 2008, p. 339), and of an even smaller number who participated in paramilitary activity (Alison, 2004). Anne recounts being sexually abused by the officer in command of her unit in the IRA. It happened when she went with her comrades to a safehouse in the Republic of Ireland for a training camp, and the officer told her to sleep in the living room with him and the other men. At night he forced her to have sex. Anne tells us she did so much give in to get it over with; she gave up, “[my] whole being sank in disgust, pain, embarrassment, grief, anger, frustration.” According to Anne, she had felt as if he was using her to demonstrate his power to the other men. To Anne, there are no words to describe the fear and trauma she experienced that night. And later when being interrogated by the police, Anne's involvement with the commanding officer was used to intimidate her. They said: “we are going to tell his wife.” Although Anne had kept quiet about the sexual relationship with the commanding officer, everyone knew.

These testimonies reflect on the heteronormative gendered discourse. That is, Karen had to just do what the men told her, without being able to rebut, or defend herself; and Anne was forced to give in and have sex with her commander in the IRA, as resisting him could have consequences for her position in the organization and for her safety. While for the officer in question, the fact that the sexualized abuse became known only attributed to his authority via the confirmation of his heteronormative and hegemonic masculinity, for Anne, the shame of carrying on a relationship with a married man meant that silence was the only possible survival strategy. The testimonies of sexualized violence illustrate Kloß's (2016) definition of sexualized violence instead of sexual violence: this term speaks to the gendered-ness of this violence and its work as a mechanism and discourse of men's control, that reasserts and reaffirms men's power. These stories highlight how it is that, that which is “not said” reveals the normalization of sexualized violence in times of conflict and postconflict peace, through silencing and hiding; and this is what must be acknowledged if the violence is to be addressed and eliminated. Therewith, the stories reflect on the heteronormative gendered discourse against which the silencing and the normalization of such silencing needs to be understood.

The testimonies of the two women outlined above point to the entrenchment of discourses on masculinity and war in the particular case of Northern Ireland. It needs to be acknowledged that speaking out against the violence would not only have meant a betrayal of the organization, but of the Irish Republican community itself. Sexualized violence, even murder, was made worse by the ethno-nationalist context, because betrayal of one's own community and the consequences of it are rarely discussed, as they are considered to be the dirty edges of war. For instance, McWilliams (1997) writes about how people were executed for passing information to the police. Within the patriarchal structures of the Northern Irish society, sexualized violence was thus silenced, and this silencing was enforced by the deep-seated divisions between ethno-/nationalist/sectarian communities in conflict. The following sections outline the various experiences that the women had with sharing their stories about sexualized violence in an artistic manner in the testimonial theater productions and community workshops, and propose how this storytelling disrupts the individual and social silence surrounding the violence.

## Finding One: Artistic Storytelling in the Theater as Transformative on an Individual Level

### Redefining a Sense of Self by Addressing the Unspeakable

The women participants in theater projects went from hiding their experiences with sexualized violence to speaking out in the theater productions, which was a cathartic and healing experience for them. As famous author, poet, and civil rights activist Maya Angelou indicates, “there is no greater agony than bearing an untold story inside of you.” This resonates with the experiences of the women participants of the Theatre of Witness project who buried the truth about their experiences with sexualized violence. We continue outlining the stories of Karen and Anne. For years, Karen had kept her experiences with sexualized violence quiet and hid her bruises from her family members, yet she believes that they must have known; nonetheless, they said nothing. In the performance, Karen describes how she not only told lies herself, but asked her children to do the same: “When we went up to Belfast with my five-year-old daughter to see my grandparents, she would ask me ‘what will we tell them, are we saying you fell down the stairs?’,—I had her telling lies too.” It was only when Karen met Teya Sepinuck, the artistic director of the Theatre of Witness, at a family support center in Derry-Londonderry, that she started to share her story about the conflict, and related experiences with sexualized violence.

Karen first shared her story with Sepinuck, and later during various preshow sessions, she shared her story with the other participants partaking in the production. In telling her story, Karen not only learned that she had a story to tell, but gained confidence to talk about her experiences with sexualized violence on the live stages of the theatrical productions and later during the community workshops. Testimonial storytelling was healing and transformative for Karen:

From the day I spoke out on stage it was a healing process for me, every time I got on the stage, it released all those skeletons one by one. It gave me a voice, one that I never had. I honestly can say that if I had not met Teya, if I had not gone on the stage to tell my story, I would not be here today. […] Through storytelling I learned to love myself.

This quote pertains to a renewed sense of agency central to the finding of one's voice (Pia, 2013), which is often lost in times of conflict (Jackson, 2002). For Karen, partaking in the Theatre of Witness production was not only a relief from the dark mental place she was in telling and sharing her story allowed her to step into the light, showing her that her story mattered and that she could use her voice and story to address the unspeakable.

The first time Anne spoke about her experiences during the conflict and the related sexualized violence was during the Theatre of Witness preshow workshops. This was an emotional and difficult experience. Anne needed to tap into her own trauma and share her previously untold story, as well as do this in front of other women, of whom some were the enemy and others had been victims of the violent actions of the IRA. (We will discuss the complexity of these conflict relations in regard to speaking out and breaking the silence in the next section). In overcoming these struggles, partaking in the Theatre of Witness production was the most cathartic experience, as Anne explains here:

In doing the Theatre of Witness project, I had learned that I was resilient and that I lived that life because it [sexualized violence] was normalized. Now, I am able to look back and talk to you about the lengths of what I have been through and say everything out loud. I now understand how Anne [referring to herself] ended up in this situation. I now know that it was not her fault.

In this quote, Anne describes her personal transformation. The quote highlights how telling her story empowered Anne to develop a new sense of self. Through this, she came to understand that what she had experienced was not her fault and that she had made choices and experienced what she did, such as the sexualized abuse, in a particular set of limited circumstances and choices. In this sense, storytelling may allow individuals to negotiate their trauma as they claim ownership over their story, name it, and with that regain agency over their experiences. As Wieskamp (2018) indicates, it is through storytelling that an individual is able to make causal connections, giving meaning to their story, as well as providing healing and transformation from past events. Narratives thus have a consoling function—to shape meaning and create an understanding of the traumatic human experiences and events in times of conflict.

In addition, we find that the experiences of Karen and Anne indicate that speaking out about experiences with sexualized violence undermines the heteronormative discourse in place, as they were able to redefine their sense of self. This, in line with Wieskamp (2018), concerns “[a] more empowered identit[y] that challenge gendered notions of victimhood” (p. 138). This can be seen in the quote by Anne, as she shifts in the way she sees herself: from a victim of sexualized violence to a survivor. According to Serisier (2020), such a shift is a main trope found in many rape memoirs, and other stories sharing about experiences with sexualized violence, and signifies how storytelling can be a site of empowerment. Anne also emphasizes this in an email sent to Jennings and Grant (2013),^2^ saying that artistic storytelling is a personal means to pull one out of victimhood and to help oneself “and others to realize just how strong, courageous and empowered one can be” (p. 77).

Henceforth, we find that through personal storytelling about sexualized violence participants are provided with an alternative language to discuss, analyze and resolve their oppression, and step out of their marginalized positions, letting their voices be heard, and redefine their sense of self and self-worth. Moreover, in tapping into the trauma of experiences with sexualized violence, participants are breaking with the persistent silence surrounding this violence. Artistic storytelling in the theatrical productions was thus a way for the participants to slay the silence within. We will now turn to how artistic storytelling in the theater may break with the social silence surrounding sexualized violence.

## Finding Two: Artistic Storytelling as Transformative on an Interpersonal and Wider Societal Level

### Telling Stories in Front of the “Other”

We see social transformation first taking place upon breaking the silence between participants partaking in the theater projects, as each shared their personal testimony of experiences during the conflict. According to the participants of the theater projects, making the productions was complex, as individuals from different sectarian backgrounds, and from different sides of the conflict, were asked to collectively reflect on the past and work toward a shared story for the theater production. These relational complexities are grounded in dualistic polarities, which stem from the sectarian structures found in society, that divided—and still divide—the people in Northern Ireland (McManus, 2017). Such dualistic polarities are inherent to cycles of violence between the good guys versus the bad guys or victims and perpetrators and can be seen in the binary between victims/survivors and perpetrators of sexualized violence. These dichotomies are important to mention as they give insight into the link between sexualized violence and the conflict and the post-conflict situation.

Anne tells us about the difficulties she faced when telling her story about the conflict and related experiences with sexualized abuse in front of the other participants. Anne feared working with the “other” (with “other” we refer to someone with an opposing sectarian or conflict background). But she was especially concerned about working with Sarah, a woman from the Catholic community (Anne's own community), whose husband was killed by the IRA:

I knew that in my production there would be a serving policewoman, so enemy, there would be a woman from the Shankill, PUL community, so enemy, there was another woman that was an enemy […]. These people wouldn’t want to be in the same room with me. There was Karen, who was from the Falls Road [Catholic/ Republican community], I believed she would be the only one there who would understand where I was coming from. And then there was Sarah, I was most afraid to meet the woman whose husband was murdered by the IRA.

Just like Anne, many participants touched upon the complex relational dynamics underpinning the interactions during the preshow workshops. Jane, a participant in the Theatre and Peacebuilding Academy production First Response (2020), recalls her hesitation to take part in the production. She was raised in a sheltered Catholic community and held very firm beliefs about the police, who were known as “scumbags,” yet, in the production, she was going to act out stories on stage with an ex-police woman. The participants questioned how they were going to create the theatrical production together.

As Anne starts to tell her story she fears the other participants to respond with anger, especially Sarah, but instead, they listened patiently and react with understanding:

I was only looking to my left side, because I was well aware of Sarah sitting on my right side. I started to tell the group what I could tell them about my story. The room was very emotional, others cried too. When I finished telling my story, I thought right I am going to turn to Sarah, whatever she has to say I am just going to take it. I turned around and right there I felt Sarah's arms wrapped around me as she gave me a big hug. ‘We are all going to be okay’, she said. I cried, she cried, there were so many tears and snotters. I didn’t expect her grace, but it had set us all up, as a group of women, to move forward, smoothly, effortlessly and with an understanding of each other without any prejudices.

It is the open and nonjudgmental environment created in the theater that allowed the women to move beyond their dualistic polarities and tell their own stories, as well as listen to the story of the “other.” Interesting to note: Anne and Sarah became close friends and are still friends to this day.

In a similar vein, Jane listened to Kelly's story and found herself feeling sympathetic and understanding of Kelly's situation and experiences. As a result, she started to let go of her own sectarian prejudices,

It was very much a learning experience. There was a police-officer, but she was very sweet. My brain didn’t understand. It was like the biggest ‘oh shit.’ It didn’t matter anymore that she was ex-police. I just learned to not let them [‘one’ sectarian side] build in prejudices, just don’t let your view be affected by the past.

In other words, through shared artistic storytelling social categories of inclusion were deconstructed. As Blagojevic (2007) explains, it is through open dialogue that social inclusion may be enhanced and relations transformed beyond conflictual or sectarian boundaries. This is illustrated by the changes on an interpersonal level, and can also be seen in a change in relations beyond the theater within family relations and the wider society. To this, we turn next.

### Sharing Stories on Stage and Beyond the Theater

This brings us to the second finding that highlights how sharing personal testimonies of abuse in artistic ways in the theater is socially transformative: it reaches audiences beyond the theater, spurring meaningful change beyond the stage. Unlike rape memoirs or the personal stories shared through the #MeToo movement, artistic stories shared in testimonial theater productions and community workshops are witnessed by a live audience. Serisier (2020) indicates that next to the act of speech, one of the most important elements in speaking out on abuse is the practice of collective listening or witnessing. We find that the audiences of both the various theater productions and community workshops, witnessing the personal stories about the conflict and related experiences with sexualized violence, are gripped by the stories told, and it encourages some to reflect upon their past experiences and give them a voice. Stories need an audience in order to be set free.

During a community workshop with Anne and Karen in 2020, reciprocal storytelling about sexualized violence took place. A young girl shared with the group of women that she had been physically and sexually harassed a few years ago by the Ulster Defense Association (UDA).^3^ The paramilitaries had infiltrated her family home, using physical force. She explained that this was the first time she had ever spoken out about her experience, but after hearing Anne's story, she felt encouraged to talk about it and break the silence. That day a group conversation unfolded about sexualized violence and the devastating effects the conflict has had on the mental health of women. Although the community workshops allowed for personal narratives about sexualized violence to be shared, it is active and engaged listening to the other that is the guiding principle that empowers individuals to tell their stories.

Aside from the community workshops, we see that telling personal stories results in reciprocal storytelling and better relationships when looking at changing relationships between family members and the participants of the theater projects. Mary, a participant in the Theatre of Witness production We Carried Your Secrets in 2009, refers to the importance of talking about the conflict, and related experiences with sexualized violence, as secrets will have devastating effects on the person keeping the secret and on the next generation. As a young girl Mary was sexually abused by her cousin. For years, she kept silent. Mary explains that it seemed that her experiences were irrelevant and disappeared in the turmoil and greater scheme of the conflict. Just as the term “The Troubles” seems to minimize and normalize the conflict, sexualized violence was similarly minimized, waved aside, and thereby normalized. Mary believes that no child should carry the weight of the secrets of their parents. She wants her son to grow up differently. She no longer wants to stay silent, instead, she will speak the truth, as the damage of keeping stuff inside will manifest in other things, cause harm, or might even result in more secrets. Anne and Karen also see how their relationship with their families changed tremendously after they shared their stories on stage during the productions. Now, their children also share their troubles with them. Telling personal stories can open up conversations of all kinds, and result in a better understanding of the other.

The evocation and empathetic testimonies in the theater may thus resonate with the experiences of the audiences (which can be seen in the aforementioned example of what happened during a community workshop), and result in reciprocal storytelling, uncovering and addressing pressing issues, such as sexualized violence. We then see that on an everyday basis, within everyday immediate relationships such as familial and friendship relations and also in the wider society, personal stories about sexualized violence need to be told to release both individuals and the collective from the silence that is manifested in the legacy of the conflict. And this is needed to bring about meaningful societal change. The concept of safe space and personal truth-telling underpin this transformative potential of artistic storytelling in these Northern Irish theater projects.

## The Theater as a Safe Space

Based on the aforementioned examples, we find that both individual storytelling and storytelling in front of the sectarian “other” and the various audiences demand safe places (see the work of Collins, 1991). This is needed so that (conflicting) narratives of identity in the margins can be produced, and hidden stories about sexualized violence can be told/shared. Central to the creation of safe space is the concept of stepping aside, which refers to the need to step “into the margins of discursive power in order to create spaces of voices from the position of difference, rather than conforming to the dominant norm” (Ghorashi and Ponzoni, 2013, p. 168). According to Ghorashi and Ponzoni (2013), such stepping aside can only occur within a nonjudgmental space, where people are able to share their stories in freedom, and in a space where there is patience for listening and producing stories close to one's own experiences.

As we explained in an earlier section of this article, in partaking in the projects the Northern Irish participants had to set aside conflictual and sectarian differences and allow disparate narratives of the violent Troubles and its aftermath to be told/shared. The theater projects were able to create a nonjudgmental space where participants freed themselves from a preconceived notion of the “other” and listened to each other's experiences, allowing disparate and hidden narratives of sexualized violence to come to the fore. In this way, the theater becomes a safe space where discursive power is negotiable and changeable, as the narratives of sexualized violence challenge and break away from the heteronormative gendered discourse and the normalized silence that surround sexualized violence.

With this in mind, we consider that artistic storytelling on sexualized violence in the theater projects may generate moral imagination (Lederach, 1997, 2005) toward a gender-just future. Coined by Lederach (2005), moral imagination refers to the capacity of individuals to creatively navigate conflict-torn societies and meander pathways toward a peaceful and humane horizon. Key to the mobilization of such moral imagination is the vision of relationships between people from different sides of deeply divided societies, in order to reflect on the past and build a better future (Lederach, 2005). As the Northern Irish women were able to transcend deep-seated conflictive and sectarian differences amongst themselves and speak up against sexualized violence, we find that the theater is a safe space that speaks to a collective imagining of a future where sexualized violence is addressed and (potentially) eliminated.

## Telling and Sharing Personal Truths

This brings us to the second point we would like to make: it is the truth of artistic narratives shared that is transformational at an individual and interpersonal level. Our participants suggest that there is “no sugarcoating reality”; your words must be true in order for others to hear them. This is explained by Anne, who sees that the success of the community workshops is built on the element of individual truth of personal narratives shared.^4^ She says that the community workshops “[bring] our stories; the dirty, the rotten, the not nice, this is what has happened, […] to schools or communities,” so that people can bear witness to stories about the conflict.

For Aileen, a participant in the Theatre and Peacebuilding Academy production First Response in 2020, it is the use of “true” narratives based on personal experiences in testimonial theater projects that are pivotal to combating sexualized violence in Northern Ireland. Aileen had been sexually abused by a previous boyfriend. When she decided to share her story with her friend, whom she knew to also have experienced sexualized violence by the same man, the friend said “oh, that happened to me too, that is just what he is like.” This reaction underscores the understanding that sexualized violence was normalized as accepted men's behavior and therewith silenced. Aileen wants to break with this trivialization and normalization of sexualized violence by speaking her truth.

Speaking personal truths then becomes a way to negotiate dominant gendered discourses, and in doing so, push for a change in the way these discourses are understood. Here Aileen talks about the importance of telling personal truths:

People have to see the trauma that the conflict has caused, how it has ruined lives and changed them so that somebody can believe it. […] I don’t want to put people through trauma, but to understand how horrendous something was for someone you must experience or see that trauma in a way, may it be through artistic storytelling or anything else, but it must be real, you can’t sugarcoat it.

The use of personal narratives in testimonial theater, especially where the speaker is positioned as a personal witness to the events being described, is normally understood by audiences to be true or authentic. That is, the audience accepts the story as credible because they can see the speaker, who is stating that it happened to her, giving them a personal connection to the events. Anne explains this as follows:

It touches a different kind of nerve knowing that the person who is telling the story has lived the story. This opens people up to the idea to tell their stories up on stage, [so] they start to reflect upon themselves. There is something more impactful when people tell their own story on stage. […] We are sort of telling people that the only person who truly knows you is you, and therefore the only person able to tell their story on stage is you.

As can be deduced from this quote, it is important that audiences bear witness to, and listen to, real stories told about life during violent conflict, and related experiences with sexual violence, in order to understand what it was like. In line with Hume's (2009) proposition to focus on the everyday epistemologies of violence, we argue that it is the everydayness of sharing personal stories about sexualized violence in the theater that is transformational. According to Anne, the actual conversation should then not merely be about sexualized violence, but about how to promote personal truth and honesty, because, as she says, “truth is the change-maker, it is the change.” To the participants of the theater projects personal truths are not only transformational themselves, but also for the audiences.

In this sense, as argued by Paula McFetridge artistic director of Kabosh Theatre Company, true narratives in the theater may pepper new-found possibilities into society, illustrating what can be achieved when one listens, hears the story of the other, and engages with it. This is because personal “true” narratives show people that their story exists. As Maria Estrada Fuentes has commented during a panel discussion on the production The Shedding of Skin by the Kabosh Theatre Company in 2021, engaged listening allows for a willingness to be undone by both the performers and audience members. Then, through the process of witnessing, the personal stories told may resonate with the individual experiences of the audiences and feed into perspectives on sexualized violence, herewith contributing to a rethinking of the social stigmas of this violence in society. In this way, testimonial theater productions and workshops are a unique tool to subvert individual and collective silence and to break with existing gendered power relations in a society that normalizes sexualized violence and silence surrounding it. As the theatrical reality works its effects on both performer and audience, artistic storytelling in testimonial theater projects is a research method that needs further investigation and development.

## Finding Three Artistic Storytelling as a Method of Research and as Actionable Knowledge

We consider two reasons why artistic storytelling in testimonial theater is epistemologically transformational. First of all, it is a new way to reiterate knowledge about experiences with sexualized violence, as well as negotiate social knowledge through the art of storytelling. Generally speaking, stories provide new ways of “re-reading and rewriting old knowledge, uncovering new narratives and locations of subjectivity,” and therefore, “stories construct social knowledge” (Clark, 2009, p. 51). With this in mind, we see that the theater is a space where knowledge is shared and communicated through performances. We consider this to be a process aligned with what Quiroga (2015) calls “co-creation of knowledge,” in this instance through storytelling in theatrical spaces.

We exemplify this by elaborating on Jane's thoughts and experiences in participating in the First Response production of the Theatre and Peacebuilding Academy in 2020. Jane describes to us how a young man grabbed her and pushed her against a car on a night out. It seemed that the boy was trying to impress his friends; “he was trying to be the big lad,” she explains. One of Jane's male friends intervened and helped her getaway. According to Jane, such a situation in Northern Ireland is not considered weird, “guys grabbing your ass, shouting stuff at you, it is so prevalent.” Yet, instead of talking about experiences with sexualized violence, it is brushed aside against a persistent heteronormative discourse that dictates that women are sexually available when they are too drunk to say no. Jane wants to break with the lingering effects of the conflict and focus on sexual assault, mental health, and sex education. It was through the First Response production that Jane and other young participants were able to share their stories about life in the aftermath of the conflict. In this sense, the play was utilized to reconstruct social knowledge, and, to use Jane's words, “steer the conversation in a way, from ‘yes, we have issues from The Troubles, to[wards] they are still affecting us today.”

This brings us to the second argument, namely that personal testimonies of violence shared in the theater not only make subjugated knowledge visible to participants and its audiences, but that the testimonies may function as actionable knowledge. In line with Erel et al. (2017), who talk about participatory theater and societal change, we see that testimonial theater projects allow participants to voice and embody their own versions of their experiences, not prompted by researchers’ questions, but rather by a collective process of reacting to other participants’ stories and experiences. In this way, participants can write their own narratives about social life, and so participants and audiences can explore the potential for change together. Returning to the above example: just as Jane pushed the narrative of the conflict forward by discussing sexualized violence and its aftermath, we find that the personal stories shared (may) challenge popular epistemologies of violence and conflict or those that are based on gender stereotypes and unequal power relations. It is especially the everydayness of the personal stories that are also epistemologically transformational, giving way to meaningful change.

Another way the theatrical productions can share and generate actionable knowledge is by continuing to share personal testimonies of sexualized violence. Anne and Karen, and other participants of the Theater of Witness project do this with the community workshops. Furthermore, together with other participants both men and women, Anne and Karen are active within the United Nations (UN) peacebuilding network. They shared their stories during a UN peacebuilding conference and did so again in 2021 through an online theatrical performance. Also, the knowledge contained within and shared through personal testimonies can produce actionable knowledge through the building of a network between artists and scholars. The discussion of the production The Shedding of Skin was a good example of this. Personal storytelling in testimonial theater projects then addresses knowledge about sexualized violence and allows participants and audiences to see their social world as one that can be changed.

With that said, we argue that personal storytelling in testimonial theater also acts as a model of practice-based research (Nelson, 2013). Practice-based research, simply stated, “is an original investigation undertaken in order to gain new knowledge, partly by means of practice and the outcomes of that practice” (Candy and Edmonds, 2018, p. 63). In the case of testimonial theater, the practice of storytelling about personal experiences with sexualized violence is a way to articulate new knowledge or actionable knowledge that may inspire and generate a change in the way the violence is understood against the dominant discourse and culture of silence. Therewith, artistic storytelling about sexualized violence could form part of arts-based research as this form of storytelling can be utilized as a mode of social research on sexualized violence, and thus produce transformative and actionable knowledge—not only for the participants, but by and with the participants.

## Critical Reflection on Artistic Storytelling

Although artistic storytelling in testimonial theater productions and community workshops has a transformative potential to address sexualized violence and rethink social stigmas, critical reflection is needed as there are some potential pitfalls to artistic storytelling in the theater. The first point of reflection is that first-person staged narratives can be more traumatizing than second-person staged narratives. As Grant (2016) explain, the biggest critique of the workings of the Theatre of Witness project is that it commodifies other people's sufferings, also referred to as “Troubles Porn,” and therefore has the potency to retraumatize the performers and the audiences. This is due to the fact that the participants step into a dual role of both witnesses and actors. In this way, the process of storytelling goes against the creative need to reflect on, challenge, and reinvent stories. In using actors to tell the stories about sexualized violence on stage, McFetridge, artistic director of the Kabosh Theatre Company, emphasizes that the performances allow for a creative process to circumvent stories. She believes theatrical productions are able to challenge the person who originally thought they owned the story, or idea, because it is their memory; and that it can do so without traumatizing them.

We consider it important to take into account the agency of the performers and the audiences, to consider if and to what extent the productions may retraumatize individuals partaking in and witnessing the production. Although some participants of the Theatre of Witness project found it retraumatizing to repeatedly tell their stories on stage, for most participants it was a cathartic experience and crucial to allow healing to take place. Anne tells us she thinks that revisiting the traumatizing past is crucial to heal, because if we don’t face trauma “it will manifest [in] illness, stress, depression, anxiety, our coping mechanisms, etc.” According to Anne, the personal input from the participants is seen as the best “counseling without counseling.” The audiences’ responses mostly echo the same sentiment. To many, the performances made them rethink their understanding of the conflict, themselves in relation to the conflict and society, and particularly their relationship with others in society. Also, the theater productions showed what a peaceful future may look like, and that there is no need to be imprisoned by the past any longer. Individual healing and transformation are thus subjective experiences, and the agency of participants and their motivation to participate should be considered and taken into account, as well as that of the audiences. It should be noted that psychological support is offered to the participants partaking in the theater productions and community workshops, and a therapist is present during the live shows.

The second concern with first-person staged narratives is that it might not generate an authentic audience response. McFetridge, when talking about the various Theatre of Witness and Theatre and Peacebuilding Academy projects, problematizes how staged narratives told by the people themselves may only give way to audience sympathy, described as a mere surface emotion that does not require interrogation of the subject discussed. She believes that the second-person staged narratives shared by actors on stage may allow for a more empathetic reaction from the audiences, which she considers needed to bring about change. This approach arguably enables the audience's reception of the work because its fictional nature allows imaginative engagement and multiple interpretations of the material. The audience can interpret the action of the drama in a way that is safe for him or her at that moment. This may also facilitate the discussions that follow Kabosh's plays, since the audiences are not constrained by the sense that they are making a personal critique of an individual's lived experience. Nonetheless, however, the perceived authenticity and truth of the first-person narrative is a powerful tool for engaging audiences in difficult conversations. When the performers speak their truth, which may disrupt the perceptions of the audience, the personal nature of the story can add weight and credibility. This is a recognized feature of documentary theater as a genre (see Bottoms, 2006; Reinelt, 2006).

The third concern of artistic storytelling is that it only allows space for certain stories to be told. To use Anne's words: “when you open a can of worms, unless you pour them all out, you can only see the ones at the top.” Although the theater productions and community workshops only reach a specific group of individuals, that is, those who are willing to speak out about their experiences or are interested in the topic, we did find that the Theatre of Witness productions and community workshops are able to reach out to individuals in various communities, and particularly hard-to-reach communities, and therefore is quite successful in facilitating change. To Anne, after 12 years, the success of these workshops is evident; they communicate and advocate for change, and encourage dialogue and actions for change. In doing so, the participants wish to bring the workshops to more schools and communities, so that more individuals are able to give voice to their experiences of sexualized violence.

## Final Remarks

In this article, we critically analyzed how artistic storytelling in testimonial theater productions and workshops can be utilized to address sexual violence with the aim of eliminating this violence in Northern Ireland. We find that participants are able to break the silence by articulating their experiences with abuse in the theater, thereby redefining their sense of self: from a victim of the violence to a survivor. In this way, the stories shared not only serve a consoling function, but contest the gendered discourse in place. Furthermore, we showed that storytelling about sexualized violence in the shared theatrical space may have a positive impact on interpersonal relations between participants, and the wider social circle, as well as on the audiences. Empirical data shows that audiences feel encouraged to tap into and work through their own trauma about the conflict and related experiences with abuse after hearing other people share their stories. Additionally, we find that storytelling in the theater is a useful method not only to yield knowledge about sexualized violence, but also to produce actionable knowledge that breaks with the normalization of silence surrounding the violence, as it opens up social debate and discussion thereon. Artistic storytelling is thus transformative at an individual and interpersonal level, as well as a social intervention. In this sense, we emphasize that artistic storytelling in the theater is a worthwhile method to be integrated into socially engaged research regarding sexualized violence.

## Notes

1. IRA, also called Provisional IRA, was a republican paramilitary organization that sought the end of British rule in Northern Ireland, and the reunification of Ireland.

2. Anne has participated in various research projects; for the research of Jennings and Grant (2013), Anne provided information about her experiences with testimonial theater via email, which we reference here.

3. The UDA, also called the Ulster Loyalist Paramilitary Organization, describes itself as defending the protestant/loyalist areas in Northern Ireland by combatting Irish republicanism.

4. With the “truth” we are not saying that there is a single truthful narrative regarding the conflict, instead we acknowledge that the stories of the participants are considered truthful to each individual as they are based on personal experiences and truths. While truth is a complex concept, in this instance, the participants refer to their words being “true” to their experiences, and to their commitment to openly speaking of difficult or shameful actions and life events.
